# Impact of different magnetic materials added to silver–magnetite nanoparticles on the structural, magnetic and antimicrobial properties

**DOI:** 10.1140/epjs/s11734-022-00759-4

**Published:** 2023-01-11

**Authors:** Asmaa. A. H. El-Bassuony, W. M. Gamal, H. K. Abdelsalam

**Affiliations:** 1 Physics Department, Faculty of Science, Cairo University, Giza, Egypt; 2 Basic Science Department, Higher Institute of Applied Arts 5th Settlement, New Cairo, Egypt

## Abstract

Different magnetic materials of spinel copper and cobalt nanoferrites added to silver–magnetite nanoparticles were fabricated by a facile, low cost, and rapid auto-combustion method to form a nanocomposite. X-ray diffraction patterns and atomic force microscopy were studied for the investigated samples and confirmed their nanosize range. Adding cobalt nanoferrite to silver–magnetite (CoAF) yielded a more pronounced effect in the magnetic measurements than adding copper nanoferrite (CuAF). This result was attributed to the much higher coercivity *H*_c_ and saturation magnetization *M*_s_ (5.7-fold and 2.8-fold, respectively) of CoAF than CuAF; accordingly, the CoAF nanocomposite can be applied to a permanent magnet. Next, the operating frequencies of the nanocomposites were calculated from the magnetic measurements. The CoAF and CuAF nanocomposites were applicable in the microwave super-high-frequency C-band and the microwave super-high-frequency S-band, respectively. Both nanocomposites were ineffective against the tested fungi but showed strong antimicrobial activities against the tested Gram-positive and Gram-negative bacteria. Thus, CoAF and CuAF nanocomposites are potential antibacterial nanomaterials for biomedical applications.

## Introduction

Spinel nanoferrites with structure formula AB_2_O_4_, where A is a divalent metal ion (such as cobalt and copper) and B is a trivalent metal ion (such as iron), are used as magnetic materials that can be applied in many applications [1, 2]. Nanoparticle materials are prepared by various solid-state and wet methods [3, 4]. Wet methods such as the sol–gel, co-precipitation, citrate, flash, and oxalate are the most effective and well known. The present study adopts the flash auto-combustion method, which is fast and low cost.

Researchers are interested in the fabrication of different magnetic materials by different methods to study their structure and physical properties to give unique materials with the best specifications to be applied in many fields. It is well known that copper (CuFe_2_O_4_) and cobalt (CoFe_2_O_4_) nanoferrites are different magnetic materials which are paramagnetic (soft) and ferromagnetic (hard) magnetic materials, respectively [5, 6].

Interest in biomedical applications has spiked since the onset of the COVID-19 pandemic. Scientists worldwide are now cooperating in the development of antiviral treatments. The most effective agents will be active against viruses, bacteria, and fungi. Bacteria and fungi are important because the misuse of antibiotics has led to strains with high resistance to the present antibiotics. Thus, the authors expect today’s antibacterial and antifungal drugs to become ineffective. An alternative nanomaterial that acts against various bacteria and fungi is imminently needed. Diamagnetic silver nanoparticles (Ag) are more effective in the nanoscale range and are known for their antiviral, anticancer, and antibacterial activities [7–11]. They are also used in water purification, biosensing, and optoelectronics devices [12–14]. Moreover, it is reported that the strongest magnetic material in the transition metal oxides is magnetite [15]. It has strong efficacy against antimicrobial properties, especially when added to silver nanoparticles [16]. Thus, in the present study, a mixture of different magnetic materials added to silver–magnetite nanoparticles was studied to give a new material with unique properties in many technological applications.

Finally, additional ferromagnetic (cobalt) and paramagnetic (copper) elements were introduced to silver–magnetite nanoparticles, and their effects on the silver–magnetite particles were studied to produce various nanocomposites. The resulting nanocomposites with concentration 0.5, 0.5 cobalt nanoferrite/0.5 silver–magnetite (0.5 CoFe_2_O_4_/0.5 Ag–Fe_3_O_4_) (CoAF) and 0.5 copper nanoferrite/0.5 silver–magnetite (0.5 CuFe_2_O_4_/0.5Ag–Fe_3_O_4_) (CuAF) were more enhanced than those in previous studies [17], and the enhancement was greater in CoAF than in CuAF. The present work investigates the structural, magnetic, and antimicrobial properties of the CoAF and CuAF nanocomposites that can be applied in many technological applications.

## Experimental details

### Sample preparation

**Fig. 1:**
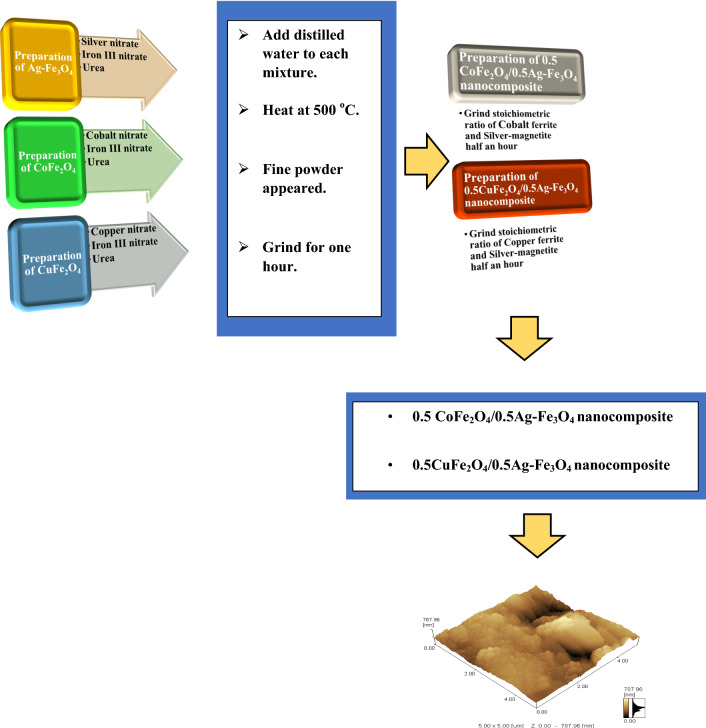
Flowchart of the flash method for fabricating CoAF and CuAF nanocomposites

Figure 1 shows the preparation method of the CoAF and CuAF nanocomposites, which is based on the flash auto-combustion technique. First, the silver–magnetite nanoparticles (Ag–Fe_3_O_4_) were prepared by mixing the metal nitrates (silver nitrate and iron III nitrate) with urea at the stoichiometric ratio and a small amount of distilled water for 0.5 h. This mixture was heated at 500 °C to form a fine powder ground for 1 h. Second, the flash method prepared the cobalt and copper nanoferrites. For this purpose, the metal nitrates (cobalt and iron III for cobalt nanoferrite and copper and iron III nitrates for copper nanoferrite) were mixed with urea and a small amount of distilled water for 0.5 h, heated at 500 °C to yield a fine powder, then ground for 1 h. Finally, cobalt nanoferrite was added to silver–magnetite at a stoichiometric concentration ratio of 0.5: 0.5 and ground for 0.5 h to form the 0.5 cobalt nanoferrite/0.5 silver–magnetite nanocomposite (0.5 CoFe_2_O_4_/0.5 Ag–Fe_3_O_4_) (CoAF). The 0.5 copper nanoferrite/0.5 silver–magnetite nanocomposite (0.5 CuFe_2_O_4_/0.5Ag–Fe_3_O_4_) (CuAF) was prepared similarly.

### Characterization

The samples were characterized by X-ray diffraction (XRD) (Diano Corporation), Fourier transforms infrared spectroscopy (FTIR) (Jasco FTIR 300 E Spectrometer), and atomic force microscopy (AFM) (Non-Contact Mode of Wet–SPM-9600). Magnetic measurements were taken on a Lake Shore 7410 magnetometer.

### Antimicrobial measurements

The Kirby method measured the antimicrobial properties in vitro [18]. The tested bacteria were the Gram-positive species *Bacillus subtilis* (ATCC 6051), *Staphylococcus aureus* (ATCC 12600), *Streptococcus faecalis* (ATCC 19433) and the Gram-negative species *Escherichia coli* (ATCC 11775), *Pseudomonas aeruginosa* (ATCC 10145), and *Neisseria gonorrhea* (ATCC 19424). The tested fungi were *Candida albicans* (ATCC 7102) and *Aspergillus fulvous* (ATCC9643). The inhibition zone diameters were measured after incubating the samples at 30 °C for 24–48 h. Statistical analysis was required for the result using ANOVA and post-hoc Turkey test to compare between the groups. The data are represented as mean value and standard deviation. The significance of the *P* value was considered when it was equal to or less than 0.05.

## Results and discussion

### X-ray diffraction pattern analysis (XRD)

**Fig. 2:**
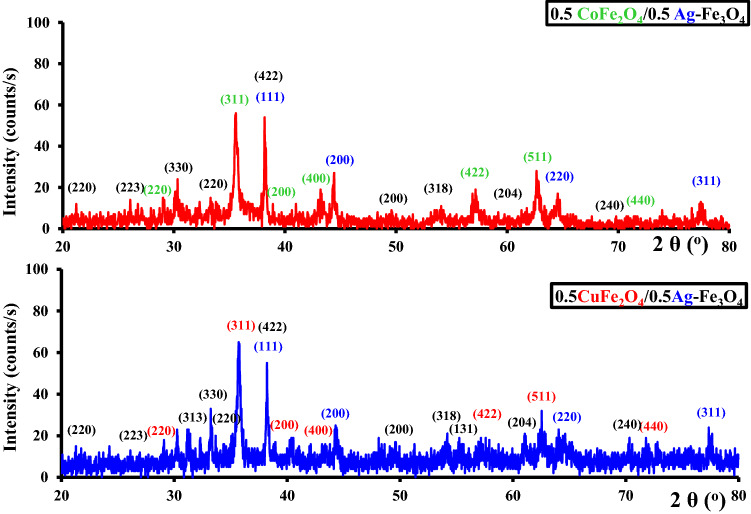
XRD patterns of the CoAF and CuAF nanocomposites

Figure 2 shows the XRD patterns of the CoAF and CuAF nanocomposites. The diffraction peaks were indexed to ICDD card numbers (04-004-6436) for silver nanoparticles, (01-084-9338) for magnetite, (04-005-7078) for cobalt nanoferrite, and (00-006-0545) for copper nanoferrite. The broadness of the XRD peaks evidenced the small sizes of the investigated samples. The crystallite sizes (see Table 1) were calculated using the following Debye–Scherrer equation [19–21]:

1 $$D = \frac{{K{ }\lambda }}{{B{\text{ cos }}\theta }}$$

where *K* is the shape factor (0.9 in the present case), *B* is the full width at half maximum, and *λ* is the wavelength of Cu-kα (1.54 Å). The crystallite size of the investigated samples is also reported in Table 1 and shows that the investigated samples are in the nanoscale range.

**Table 1:** Crystallite sizes from XRD patterns, particle sizes obtained from AFM images, roughness, unit cell parameters, and volume parameters of the CoAF and CuAF nanocomposites

Sample	Crystallite size (nm)	Particle size AFM (nm)	Roughness (μm)
0.5CoFe_2_O_4_/0.5Ag–Fe_3_O_4_	59.1	147.49	1.27
0.5CuFe_2_O_4_/ 0.5Ag–Fe_3_O_4_	44.3	73.83	1.41

### Fourier transform infrared analysis (FTIR)

**Fig. 3:**
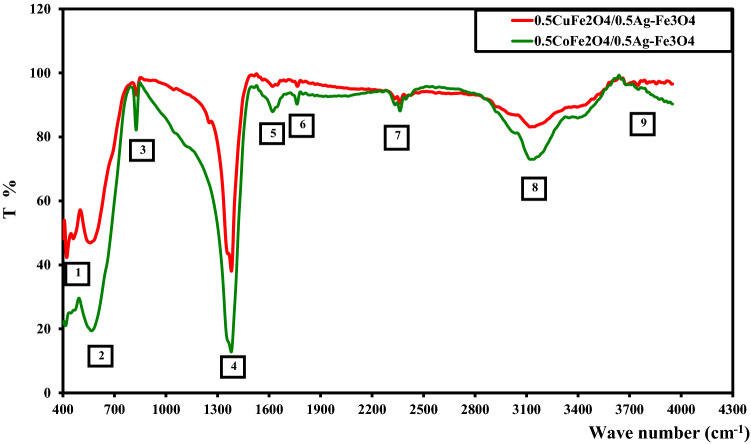
FTIR spectra of the CoAF and CuAF nanocomposites

Figure 3 and Table 2 show the FTIR spectra of the CoAF and CuAF nanocomposites in the range 400–4000 cm^−1^. Peaks 1 and 2, which appeared in the spectra of all ferrites, were attributed to stretching vibrations of the metal–oxygen bonds at the octahedral B sites and tetrahedral A-sites, respectively [22]. Peak 3 was contributed by water molecules. Peaks 4 and 5 were attributed to C–O–C vibrations or possibly to C=N vibrations from urea [23]. Peak 6 was assigned to stretching vibrations of the OH group. Finally, peaks 7 and 8 were contributed by stretching vibrations of the OH and NH groups, respectively. All FTIR results were consistent with those of previous works [24]. Thus, FTIR analyses assured the formation of the investigated nanocomposite.

**Table 2:** FTIR results of the CoAF and CuAF nanocomposites

No. of peaks	1	2	3	4	5	6	7	8
0.5CoFe_2_O_4_/0.5Ag–Fe_3_O_4_	415.6	566.0	826.3	1620.9	1762.6	2362.4	3138.6	3708.4
0.5CuFe_2_O_4_/0.5Ag–Fe_3_O_4_	422.3	558.3	828.3	1620.8	1765.5	2362.3	3121.2	3747.0

### Atomic force microscopy analysis (AFM)

**Fig. 4:**
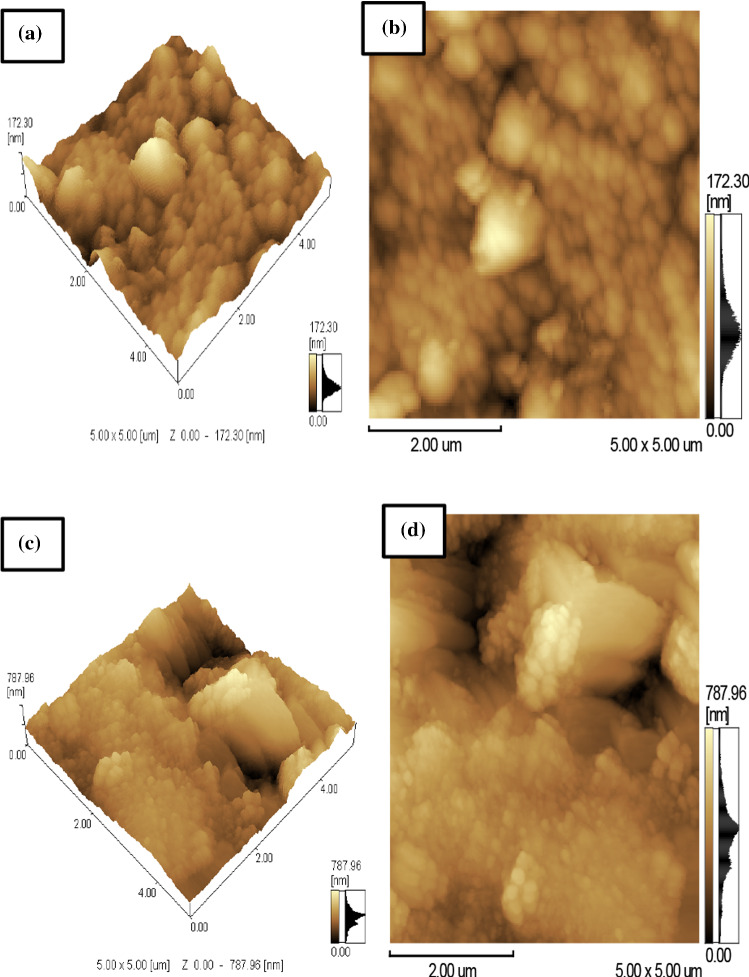
AFM micrographs and plane images of the CoAF and CuAF nanocomposites

**Fig. 5:**
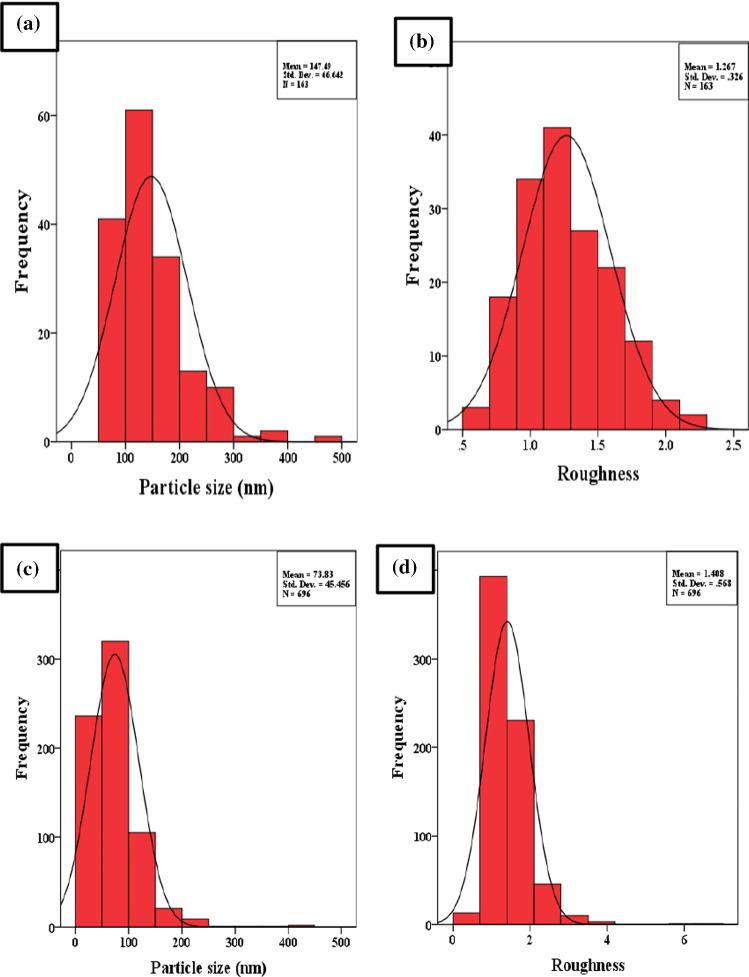
Histograms of average particle sizes and roughness values of the CuAF nanocomposite

Figure 4a–d shows the three-dimensional and plane images of AFM micrographs of the CoAF and CuAF nanocomposites. It is observed from the micrographs that agglomeration occurred for both samples because no surfactant was applied during the preparation method [25, 26]. Figure 5a–d shows the histogram of the average particle size and the roughness extracted from the AFM micrographs using the IBM SPSS Statistics 22 program. One can observe from Table 1 that the average particle size of CoAF is larger than that of CuAF nanocomposite, and this result is confirmed by XRD analysis. In addition, the average particle size of the CoAF and CuAF nanocomposite confirmed the nanosize range of the nanosamples, as shown in Table 1 and Fig. 5a, c. Moreover, Fig. 5b, d shows that CuAF nanocomposite had larger roughness than CoAF nanocomposite. This was attributed to the larger surface activity of CuAF nanocomposite than that of CoAF nanocomposite. As a result of XRD and AFM analyses, the XRD patterns yielded a smaller size than the AFM measurements because AFM obtains aggregates of small crystallites, whereas XRD measures the individual crystallites.

### Magnetic measurements

**Fig. 6:**
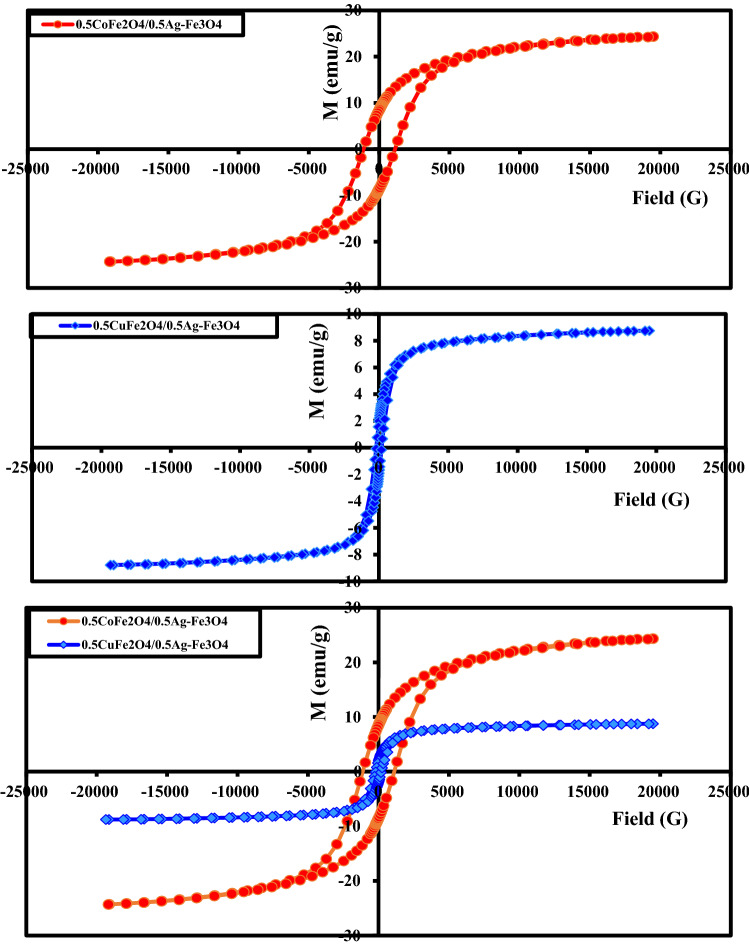
Magnetic hysteresis loops of the CoAF and CuAF nanocomposites at 300 K

Figure 6 shows the magnetic hysteresis loops of CoAF and CuAF nanocomposites at room temperature (300 K) under a maximum applied field of 20 KG. All samples showed ferromagnetic behavior. Table 3 reports the magnetic parameters of the samples, namely, the coercivity (*H*_c_), saturation magnetization (*M*_s_), remanent magnetization (*M*_r_), squareness (*R*), and magnetocrystalline anisotropy constant (*k*). The coercivity of the CoAF nanocomposite was 5.7-fold larger than that of CuAF (Fig. 6 and Table 3), implying that CoAF can be applied as a permanent magnet. The large *H*_c_ was attributed to the 15.8-fold higher magnetocrystalline anisotropy constant of the CoAF nanocomposite than the CuAF nanocomposite. The *k* was calculated as [27, 28]

2 $$k = \frac{{M_{{\text{s}}} H_{{\text{c}}} }}{0.98}$$

**Table 3:** Magnetic constants of the CoAF and CuAF nanocomposites

Magnetic parameters	*H*_c_ (G)	*M*_s_ (emu/g)	*M*_r_ (emu/g)	Squareness (*M*_r_/*M*_s_)	Magnetocrystalline anisotropy constant *k* (erg/g)	Magnetic susceptibility *χ* (emu/g. G)	*ω* GHz)
0.5CoFe_2_O_4_/0.5Ag–Fe_3_O_4_	1117.3	24.333	8.5224	0.35023	27,742.1	0.00913	5.4
0.5CuFe_2_O_4_/0.5Ag–Fe_3_O_4_	196.59	8.7642	1.9415	0.22152	1758.1	0.0101	1.9

Moreover, the saturation magnetization *M*_s_ was 2.8-fold higher in the CoAF nanocomposite than in the CuAF nanocomposite. This result can be explained by the different magnetic behaviors of the nanocomposites: the cobalt in CoAF is ferromagnetic, whereas the copper in CuAF is paramagnetic. As reported in previous work, materials with high *M*_s_ confer high antimicrobial properties [37]. As discussed below, the samples investigated in this study also showed strong antimicrobial activities. The squareness *R* was calculated as [29, 30]

3 $$R = \frac{{M_{{\text{r}}} }}{{M_{{\text{s}}} }}$$

As shown in Table 3, the squareness was 1.6-fold larger in the CoAF nanocomposite than in the CuAF nanocomposite. This result is directly related to the remanent magnetization, which was 4.4-fold higher in the CoAF nanocomposite than in the CuAF nanocomposite as the squareness was below 0.5 in both samples the particles interacted by magnetostatic interactions.

**Fig. 7:**
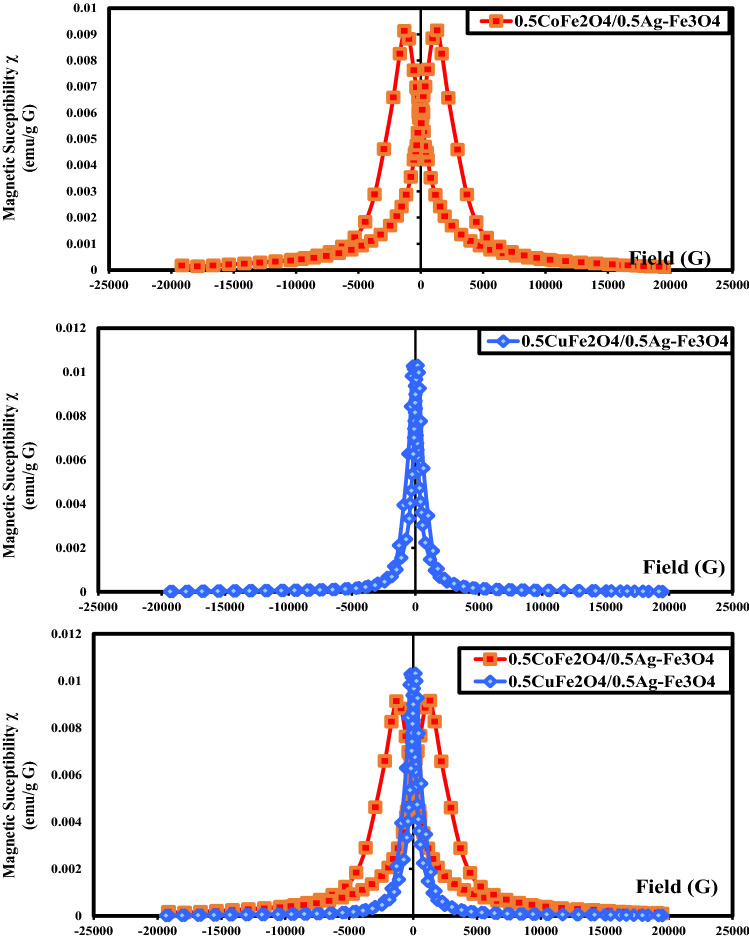
Magnetic susceptibility diagrams of the CoAF and CuAF nanocomposites

Figure 7 shows the field dependences of the magnetic susceptibilities of the CoAF and CuAF nanocomposites. The magnetic susceptibility (*χ*) was calculated by differentiating the magnetization to the applied field [31]:

4 $$\chi = \frac{dM}{{dH}}$$

The values are reported in Table 3. The very large *χ* values under zero-field well agreed with those of a previous report [32]. The *χ* was 1.1-fold higher in the CuAF nanocomposite than in the CoAF because the latter undergoes inefficient exchange coupling between the soft and hard magnetic phases [33, 34]. However, the width was larger in the CoAF nanocomposite than in the CuAF nanocomposite, again owing to the 5.7-fold higher *H*_c_ of the CoAF nanocomposite than the CuAF nanocomposite.

### High-frequency application

**Fig. 8:**
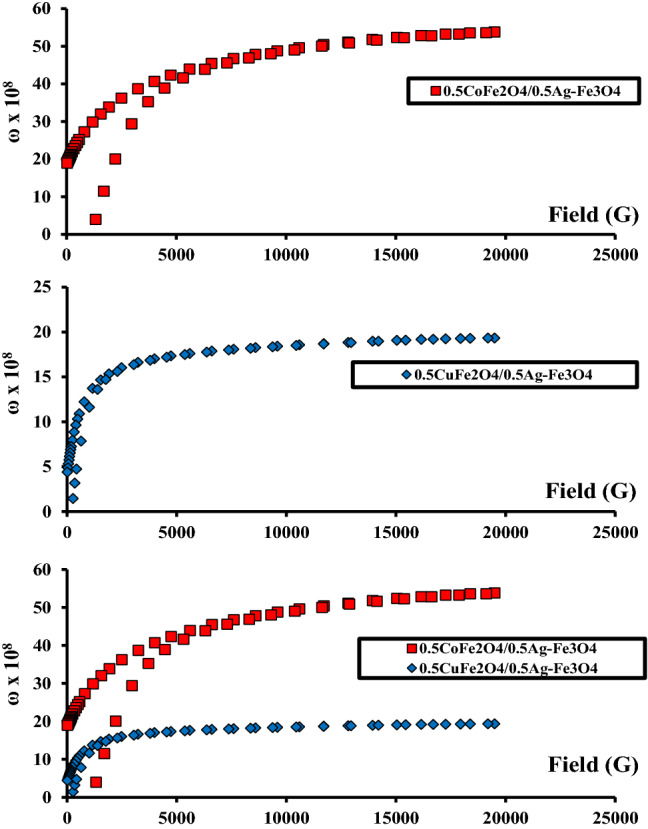
Operating frequency diagrams of the CoAF and CuAF nanocomposites

The operating frequencies (*ω*) of the CoAF nanocomposite were obtained from magnetic measurements [35] and are shown in Fig. 8. The operational frequency was estimated as follows [36, 37]:

5 $$\omega = 8\pi^{2} \gamma M$$

where *γ* = 2.8 MHz/G is the gyromagnetic ratio. Table 3 summarizes the results. The operating frequency of the CoAF nanocomposite was 2.8-fold higher than that of the CuAF nanocomposite, indicating that the CoAF nanocomposite has a 2.8-fold higher *M*_s_ than the CuAF nanocomposite. Both samples seem to have potential in a variety of applications. The CoAF and CuAF nanocomposites’ operating frequencies (5.4 and 1.9 GHz, respectively; see Table 3) could be applied in the microwave super-high-frequency C-band and microwave super-high-frequency S-band. The operating frequencies help evaluate the performance of nanodevices that operate in the high-frequency bands. It’s worth noting that the operating frequency is influenced by both the device’s saturation magnetization and its shape. It is reported that microwave radiation with frequencies from 0.3 to 300 GHz damage the microbial cultures [38]. Thus, it is strongly recommended to apply the present study nanocomposite in microwave super-high frequency and use it to kill different bacteria.

### Antimicrobial study

**Fig. 9:**
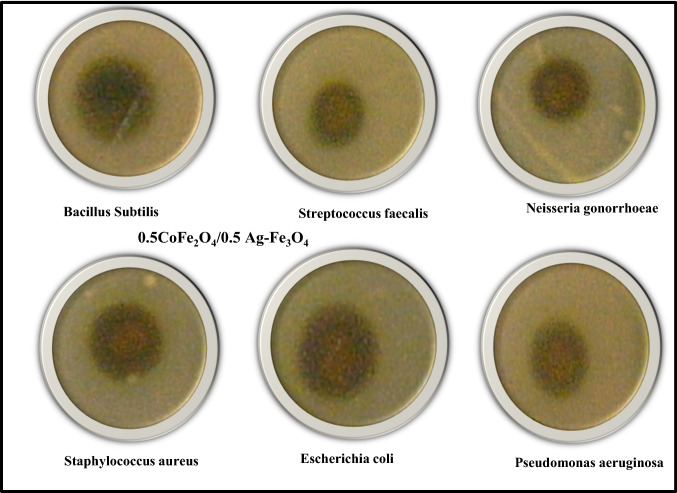
Antimicrobial activities images of the CoAF nanocomposite

**Fig. 10:**
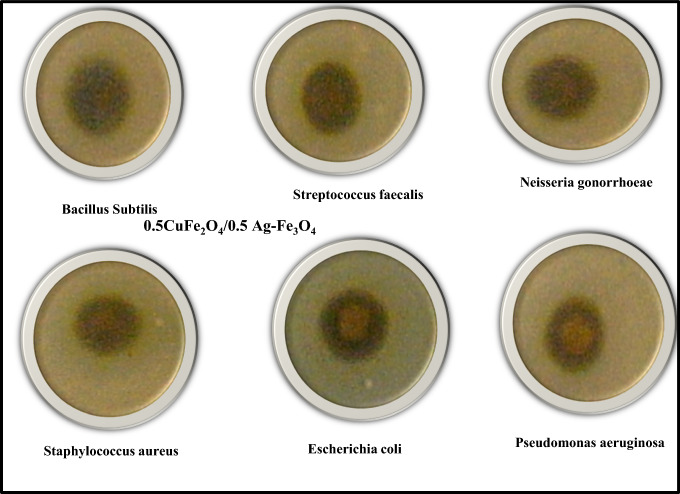
Antimicrobial activities images of the CuAF nanocomposite

**Fig. 11:**
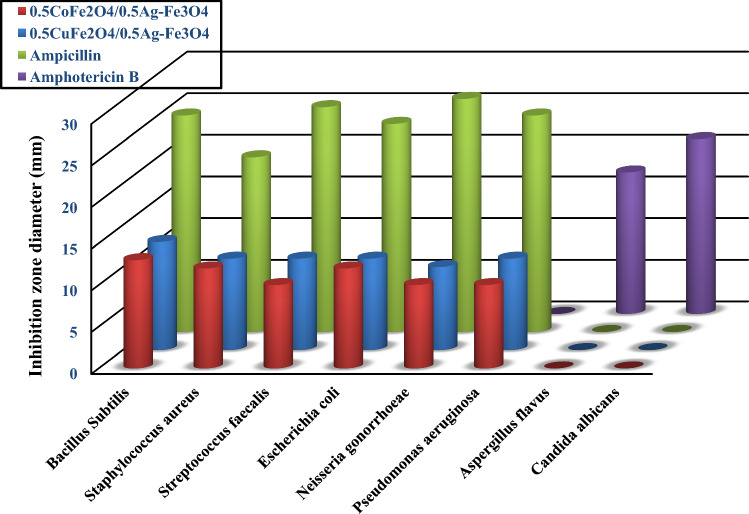
Antimicrobial activity comparisons of the CoAF and CuAF nanocomposites and standard antibacterial and antifungal agents

The antibacterial and antifungal activities of the CoAF and CuAF nanocomposites are shown in Figs. 9, 10, and 11. The bacteria that were examined included both Gram-positive and Gram-negative species, as can be seen in Table 4. Moreover, Table 4 shows the data collected as mean and standard deviation values, and statistics were done to assure that there is a significant difference between the investigated samples in which the *P* value was less than 0.05. The different lowercase letters in the same row indicate that there is a significant difference (*P* < 0.05). However, the similar lowercase letter between the row indicates that there is no significant difference (*P* > 0.05). The investigated samples demonstrated strong activity against both bacterial types (Gram-positive and Gram-negative) because silver nanoparticles destroy the DNA of bacterial cell membranes [39–42]. However, both samples did not work against the tested fungi. It is clear from looking at the numbers that the activity that the CoAF nanocomposite exhibited against the microorganisms *Staphylococcus aureus* and *Escherichia coli* was much higher than that of the CuAF nanocomposite. CuAF nanocomposite, on the other hand, had a higher level of effectiveness against *Streptococcus faecalis* and *Pseudomonas* *aeruginosa* bacteria. As a result, the investigated nanomaterials are strongly recommended to be used as alternative antibacterial nanomaterials for various drugs.

**Table 4:** Antimicrobial parameters of the CoAF and CuAF nanocomposites and standard antibacterial and antifungal agents

Samples	Inhibition zone diameter (mm)							
	Bacteria						Fungi	
	G +			G-				
	*Bacillus subtilis*	*Staphylococcus aureus*	*Streptococcus faecalis*	*Escherichia coli*	*Neisseria gonorrhoeae*	*Pseudomonas aeruginosa*	*Aspergillus flavus*	*Candida albicans*
0.5CoFe_2_O_4_/0.5Ag–Fe_3_O_4_	13 ± 0.2a	12 ± 0.5b	10 ± 0.3c	12 ± 0.5b	10 ± 0.3c	10 ± 0.3c	0 ± 0.0d	0 ± 0.0d
0.5CuFe_2_O_4_/0.5Ag–Fe_3_O_4_	13 ± 0.2a	11 ± 0.5e	11 ± 0.5e	11 ± 0.5e	10 ± 0.3c	11 ± 0.5e	0 ± 0.0d	0 ± 0.0d
Ampicillin	26 ± 0.2f	21 ± 0.2g	27 ± 0.3h	25 ± 0.2i	28 ± 0.2j	26 ± 0.2f	0 ± 0.0d	0 ± 0.0d
Amphotericin B	0 ± 0.0d	0 ± 0.0d	0 ± 0.0d	0 ± 0.0d	0 ± 0.0d	0 ± 0.0d	17 ± 0.2k	21 ± 0.2g

The same lowercase alphabets (a–k) are insignificantly different (*P* > 0.05), however those with different ones are significantly different (*P* > 0.05)

## Conclusion

CoAF and CuAF nanocomposites were successfully prepared by a facile flash method. The structural characterizations confirmed the nanoscale range of the particle sizes. Meanwhile, the magnetic properties showed a 5.7-fold and 2.8-fold higher coercivity and saturation magnetization, respectively, in CoAF than in CuAF nanocomposite, implying that CoAF is a permanent magnet material. The operating frequencies of both samples showed that CoAF and CuAF could be applied in the microwave super-high-frequency C-band and the microwave super-high-frequency S-band, respectively. Finally, both samples showed strong antibacterial efficacy against the tested Gram-positive and Gram-negative species. Thus, they are potentially recommended to be used as antibacterial nanomaterials in biomedical applications.

## Data Availability

The data that support the findings of this study are available on request from the corresponding author.
